# The effect of the genomic GC content bias of prokaryotic organisms on the secondary structures of their proteins

**DOI:** 10.1371/journal.pone.0285201

**Published:** 2023-05-04

**Authors:** Diana Barceló-Antemate, Fernando Fontove-Herrera, Walter Santos, Enrique Merino

**Affiliations:** 1 Departamento de Microbiología Molecular, Instituto de Biotecnología, Universidad Nacional Autónoma de México, Cuernavaca, Morelos, México; 2 Centro de Investigación en Dinámica Celular, Instituto de Investigación en Ciencias Básicas y Aplicadas, Universidad Autónoma del Estado de Morelos (UAEM), Cuernavaca, Morelos, México; 3 C3 Consensus, León, Guanajuato, México; Boyce Thompson Institute, UNITED STATES

## Abstract

One of the main characteristics of prokaryotic genomes is the ratio in which guanine-cytosine bases are used in their DNA sequences. This is known as the genomic GC content and varies widely, from values below 20% to values greater than 74%. It has been demonstrated that the genomic GC content varies in accordance with the phylogenetic distribution of organisms and influences the amino acid composition of their corresponding proteomes. This bias is particularly important for amino acids that are coded by GC content-rich codons such as alanine, glycine, and proline, as well as amino acids that are coded by AT-rich codons, such as lysine, asparagine, and isoleucine. In our study, we extend these results by considering the effect of the genomic GC content on the secondary structure of proteins. On a set of 192 representative prokaryotic genomes and proteome sequences, we identified through a bioinformatic study that the composition of the secondary structures of the proteomes varies in relation to the genomic GC content; random coils increase as the genomic GC content increases, while alpha-helices and beta-sheets present an inverse relationship. In addition, we found that the tendency of an amino acid to form part of a secondary structure of proteins is not ubiquitous, as previously expected, but varies according to the genomic GC content. Finally, we discovered that for some specific groups of orthologous proteins, the GC content of genes biases the composition of secondary structures of the proteins for which they code.

## Introduction

In prokaryotes, the guanine and cytosine composition (GC content) of genomic DNA has been extensively studied and has been shown to vary markedly, from below 20% to approximately 75% [1, 2]. The causes of this great difference in genomic GC content are not yet known with certainty, but it has been correlated with genomic size [3, 4], lifestyle [5, 6], environmental habitats [7], environmental conditions [8–10], and mutational pressure [11–13], among other variables.

Because the genetic code is degenerate [14, 15], there are amino acids, such as Ala, Gly, Pro, and Arg, that are encoded by GC-rich codons, while others, such as Lys, Asn, and Ile, are encoded by AT-rich codons [13, 16, 17]. It is well known that not all codons are equally used by organisms; rather, there is a preference for codon usage that is linearly correlated with the genomic GC content across phyla [18, 19] and with the relative amino acid content of their corresponding proteomes [19, 20]. This impact of the genomic GC content on the amino acid composition of the proteomes has been well documented [16, 17, 21–23]. Such studies reported that genomic GC content variation is one of the main contributors to the proteomic architecture of organisms at a primary structural level, mainly affecting the frequency of those amino acids encoded by GC- or AT-rich codons [24].

The tendencies of amino acids to be part of secondary structures like alpha-helices, beta-sheets, and random coils, have been based on how frequently they are present in such structures, consistently with their physical and chemical properties [25, 26]. These tendency values are known as the amino acid conformational parameters and can be used to classify the amino acids as indifferent, former, strong former, breaker, or strong breaker secondary structure elements of proteins (S1 Table) [25, 27]. As an example, Val is considered a strong beta-sheet former and has a low tendency to be part of random coils. In any case, these tendencies of each amino acid to be part of a specific secondary structure of proteins have been idealized as invariant to all organisms.

Our study shows for the first time the impact of genomic GC content on the secondary structure of proteins and proteomes in prokaryotic organisms. Our results are presented and discussed at different levels of organization: at a taxonomic level, by analyzing the distribution of genomic GC content in different phylogenetic clades; at the proteomic level, by evaluating the impact of the genomic GC content on the relative frequencies of primary and secondary structures of proteins; at the secondary structure level of proteins, by analyzing the effect of the genomic GC content on the amino acid tendencies to be part of such elements; and on the way in which the genomic GC content influences the relative frequencies of the different secondary structures in orthologous proteins.

## Materials and methods

The sequence analysis performed in our article was executed in a hierarchical order by different units of analysis, as indicated in S2 Table, and corresponds to the following tasks:

### Genome and proteome data set

A set of 4,655 genomic sequences of nonredundant species of prokaryotes was extracted from the KEGG GENOME Database, May 2022. In this set of sequences, we evaluated the range of their genomic GC content. To avoid overrepresentation, for each integer value of genomic GC, three to four prokaryote sequences were chosen randomly, with the end of having the most uniform distribution of representative sequences among the genomic GC values. We obtained 192 organisms with genomic GC contents ranging from 20% to 74% and covered the Bacteria and Archaea domains. The amino acid sequences of the selected organisms were also obtained from the same database. In addition, 1,544 representative prokaryotes at the genus level were also characterized in terms of their genomic GC content and the secondary structure of their proteins. This second set of organisms was used in our analysis of orthologous proteins.

### Taxonomic classification

The taxonomic assignment of the organisms was carried out according to the KEGG GENOME database [28] (https://www.genome.jp/kegg/genome/).

### Genomic GC content evaluation

Considering the purpose of our study regarding the analysis of the primary and secondary structures of proteins in relation to the GC content of the DNA in which they are encoded, for the genomic GC content evaluations, we only considered the genomic regions corresponding to protein-coding genes. The genomic coordinates of the protein-coding genes were taken from the KEGG GENES database [28] (https://www.genome.jp/kegg/genes.html).

We calculated the coding region genomic GC content for each of the 192 selected organisms using the following formula:

$$GC_{G}=\frac{\sum_{g\in P\left(G\right)}GC(g)}{\sum_{g\in G}\;|g|},$$

where *G* is the genome of interest, *P(G)* is the protein-coding genes of *G* (the proteome of *G*), *g* is a given protein-coding gene, |*g*| is its length, and *GC(g)* is the number of G or C nucleotides in *g*.

### Classification of amino acids according to the GC content of their codons

**Fig 1:**
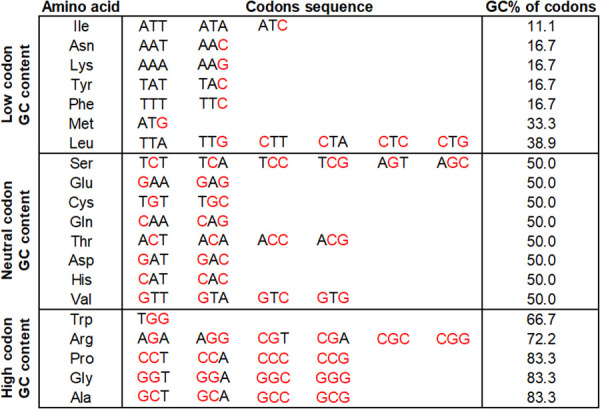
Classification of the genetic code according to codon GC content. The G and C nucleotides are highlighted in red. The GC contents of codons are expressed as a percentage in the last column. The 20 standard amino acids were classified according to the number of G or C nucleotides divided by the number of nucleotides in their respective codons.

We classified the 20 amino acids of the genetic code according to the GC content of their codons into three groups. The first group has 5 amino acids with high-GC content codons: A, G, P, R, and W; the second has 8 amino acids with neutral-GC content codons: V, H, D, T, Q, C, E, and S; and the third group has 7 amino acids with low-GC content codons: L, M, F, Y, K, N, and I (Fig 1).

### Evaluation of the amino acid frequencies in the proteomes

For every proteome of our 192 studied organisms, we evaluated the relative frequencies of each of the 20 amino acids using the following formula:

$$aa^{G}=\frac{\sum_{p\in P\left(G\right)}\;|p(aa)|}{\left|P(G)\right|},$$

where *G* is the genome of interest, *P(G)* is their proteome, *aa* is a given amino acid, *p* is a protein, |*p(aa)*| is the number of times the amino acid aa appears in protein *p*, and |*P(G)*| is the total number of amino acids in proteome *P* of *G*.

### Prediction of the protein’s secondary structure

The secondary structure of the proteins was predicted using PSSPRED [29] version 4 (https://seq2fun.dcmb.med.umich.edu//PSSpred/).

### Evaluation of the amino acid frequencies in the secondary structures of the proteomes

We analyzed the content of the amino acids in the different secondary structures of proteins of our 192 studied organisms: alpha-helices, beta-sheets, and random coils, using the following formulas:

$$aa_{\alpha }^{G}=\frac{\sum_{p\in P\left(G\right)}\;|\alpha (p,aa)|}{\sum_{p\in P\left(G\right)}\;|\alpha (p)|},$$

$$aa_{\beta }^{G}=\frac{\sum_{p\in P\left(G\right)}\;|\beta (p,aa)|}{\sum_{p\in P\left(G\right)}\;|\beta (p)|},$$

$$aa_{\gamma }^{G}=\frac{\sum_{p\in P\left(G\right)}\;|\gamma (p,aa)|}{\sum_{p\in P\left(G\right)}\;|\gamma (p)|},$$

where *G* is the genome of interest, *P(G)* is their proteome, *aa* is a given amino acid, *p* is a protein, |*ss(p*,*aa)*| is the number of times that *aa* appears in the secondary structure *ss* (may be *α*: alpha-helix, *β*: beta-sheet or *γ*: random coil) in a protein *p*, and |*ss(p)*| is the length of the secondary structure *ss* in protein *p*.

### Analysis of genomic GC content bias effects on the conformational parameters of amino acids in the secondary structure of proteins

For each of the 192 sequence proteomes considered in our study, we evaluated the amino acid conformational parameters for secondary structures using the original procedure described by Chou and Fasman [25]. It is important to describe two variables. The first is the frequencies of each amino acid residue present in the secondary structures (alpha-helix, beta-sheets, and random coils) in relation to the frequencies of such amino acids in the proteome.


$$F_{ss}^{aa}=\frac{\sum_{p\in P\left(G\right)}\;|ss(p,aa)|}{\sum_{p\in P\left(G\right)}\;|p(aa)|},$$

The second is the relative frequencies of the amino acids for each secondary structure (alpha-helix, beta-sheet, and random coil) in relation to the number of amino acids in the proteome.


$$F_{SS}=\frac{\sum_{p\in P\left(G\right)}\;|ss(p)|}{\left|P(G)\right|},$$

The conformational parameters are obtained when $F_{ss}^{aa}$ is divided by *F*_*ss*_, as seen in the following formula:

$$CP=\frac{F_{ss}^{aa}}{F_{ss}}.$$

Where *G* is the genome of interest, *P(G)* is their proteome, |*P(G)|* is its length, *aa* is a given amino acid, *p* is a protein, |*ss(p*,*aa)*| is the number of times that *aa* appears in the secondary structure *ss* (may be *α*: alpha-helix, *β*: beta-sheet or *γ*: random coil) in a protein *p*, *|p(aa)|* is the number of times *aa* appears in protein *p*, and |*ss(p)*| is the length of the secondary structure *ss* in protein *p*.

The values of the amino acid conformational parameters thus obtained were then plotted against the GC content of their corresponding genomic sequences.

### Evaluation of secondary structure frequencies of the proteomes

The frequencies in which the different secondary structure elements, alpha-helices, beta-sheets, or random coils, were represented in the proteomes were evaluated using the following formulas, respectively:

$$Alfa^{G}=\frac{\sum_{p\in P\left(G\right)}\;|\alpha (p)|}{\left|P(G)\right|},$$

$$Beta^{G}=\frac{\sum_{p\in P\left(G\right)}\;|\beta (p)|}{\left|P(G)\right|},$$

$$Coil^{G}=\frac{\sum_{p\in P\left(G\right)}\;|\gamma (p)|}{\left|P(G)\right|},$$

where *G* is the genome of interest, *P(G)* is its proteome, |*P (G)*| is the total number of amino acids in the proteome, *p* is a given protein of the proteome *P(G)*, *α(p)* is the number of amino acids located in alpha-helices, *β(p)* is the number of amino acids in beta-sheets, and *γ(p)* is the number of amino acids that are part of the random coils.

### Clustering of proteins into COG groups

From the KEGG GENOME Database [28], a set of 1,544 representative prokaryotes at the genus level were chosen for this study. The proteins of this set of organisms were clustered into COG groups (Clusters of Orthologous Genes) [30] by using a four-step computational procedure. First, for a given COG in the COG database from NCBI (https://www.ncbi.nlm.nih.gov/research/cog), we obtained all the corresponding proteins. Second, for every set of COG groups, we aligned the proteins using the MUSCLE v5 program [31]. Third, a hidden Markov model for every COG was built using the *hmmerbuild* program of the HMMER v3.3.2 package [32]. Fourth, using the HMM matrices built for every COG, we scanned all the protein sequences in our set of organisms to identify the protein domains that better corresponded to a COG HMM model.

### Selection of representative protein sequences by COGs

To select the orthologous protein sequences that best represented each COG, the distribution of the lengths of the proteins belonging to every COG was obtained, and the mean and standard deviation were evaluated. As a first criterion to consider a protein as representative of a COG for our secondary structure analysis, we only included those proteins whose lengths were located at no more than one standard deviation from the mean of the corresponding length distribution. For our second inclusion criterion, we evaluated the distribution of the lengths of the COG domains identified in the proteins using the corresponding COG’s hidden Markov models. We only included those proteins whose sequence covered at least 80% of the mean value of COG domain length.

The first condition was used to exclude multidomain proteins with large regions of sequence that were not associated with the COG to be analyzed, and the second condition was used to discard proteins with partial COG domains.

### Evaluation of the secondary structure frequencies of COG proteins

The secondary structure of the proteins of the COGs was predicted using PSSPRED [29] version 4. With these data, we calculated the content of the secondary structure: alpha-helixes, beta-sheets, and random coils for each of the 4,511 COGs in our study using the following formulas:

$$COG_{\alpha }^{p}=\frac{\left|\alpha (p)\right|}{\left|p\right|},$$

$$COG_{\beta }^{p}=\frac{\left|\beta (p)\right|}{\left|p\right|},$$

$$COG_{\gamma }^{p}=\frac{\left|\gamma (p)\right|}{\left|p\right|},$$

where *p* is a given protein, |*p*| is the length of the protein sequence, and |*ss(p)*| is the number of amino acids predicted to be present in one of the three main secondary structures of the protein (*α*: alpha-helix, *β*: beta-sheet, or *γ*: random coil).

### Multiple alignments of the secondary structure elements of orthologous proteins

The secondary structures of proteins were represented as a three-letter code, H, E, and C, to represent the alpha-helix, beta-sheet, and random coil elements. The secondary structure elements of each COG were aligned using the MUSCLE program [31].

### Programming languages used

The pipeline generated to access and process the data from the KEGG database and the results obtained by the PSSPRED program [29] were written in Perl v5.30.0, Python v3.10.10, and R v3.6.1 and are available at our web page https://biocomputo.ibt.unam.mx/gcto2d/programs/.

The analysis of the data was performed using the *Pandas* and *NumPy* packages of Python. The statistical analysis, linear regression and *p-value*, was performed using *format*.*pval* from base package of R. The *p-values* obtained for the different linear regression analyses of our study were used to test whether our data could have occurred under the null hypothesis. The codes used to represent the different *p-values*, ordered from highest to lowest, are: 0 ’***’; 0.001 ’**’; 0.01 ’*’; 0.05 ‘.’; 0.1 ‘ ‘.

We developed our GCto2D web service (https://biocomputo.ibt.unam.mx/gcto2d/) to make our results regarding the effect of the GC content of genes on the secondary structures of orthologous proteins available to the scientific community. Our GCto2D web page was developed using HTML5/CSS as the frontend and supplemented with a combination of vanilla JavaScript, PHP, Perl, and MySQL for the backend to guarantee a fast interaction with modern web browsers. The deployment service is hosted in an instance of Apache HTTPD server v2.4.

## Results and discussion

### Diversity of genomic GC content across prokaryotic phyla

**Fig 2:**
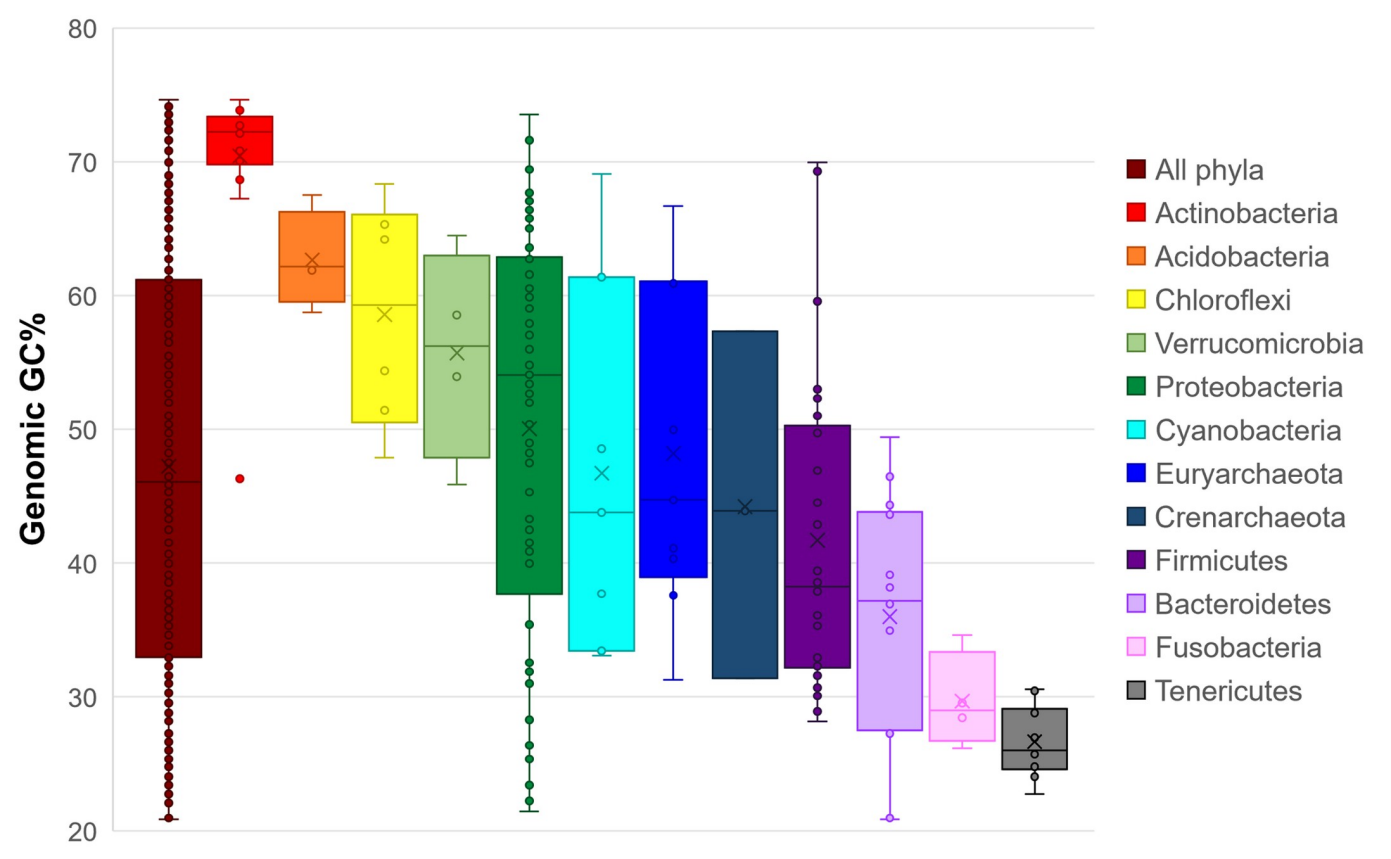
Distribution of the genomic GC content of 192 studied prokaryotes. The boxplots show the results of 12 phyla with at least three organisms with respect to their genomic GC content. The first boxplot corresponds to the genomic GC content values of all the studied microorganisms, which range from 20 to 74%. The boxplots were ordered in accordance with the GC content values of the first quartile.

From a set of 4,655 nonredundant genomic sequences of prokaryote species, we selected 192 based on their genomic GC content, 178 sequences corresponding to bacterial organisms, and 14 to archaeal organisms. The genomic GC content range of these studied organisms was wide, from 20 to 74% (Fig 2). The values of the genomic GC content of the organisms were clustered into 32 phyla, of which 28 were from bacteria and only 4 were from archaea (S3 Table). Clear trends specific to each phylum were observed. Actinobacteria was the phylum with the highest genomic GC content, with a mean of 70.42%, with members such as *Cellulomonas fimi* ATCC 484 presenting the highest GC content value of 74.6%. Acidobacteria also had a high GC content, with a mean of 62.64%. On the other hand, Fusobacteria and Tenericutes presented low genomic GC content values, with means of 29.69% and 26.64%, respectively. The phylum Proteobacteria merits special mention. It is the phylum with the largest number of representative sequenced organisms and with the widest genomic GC content range. The mean genomic GC content varied between the main four classes of Proteobacteria, Alpha, Beta, Delta, and Gamma, with values of 54.41%, 58.67%, 57.37%, and 45.53%, respectively.

### The genomic GC content imposes bias in the amino acid frequencies of the proteome and the secondary structures of their proteins

**Fig 3:**
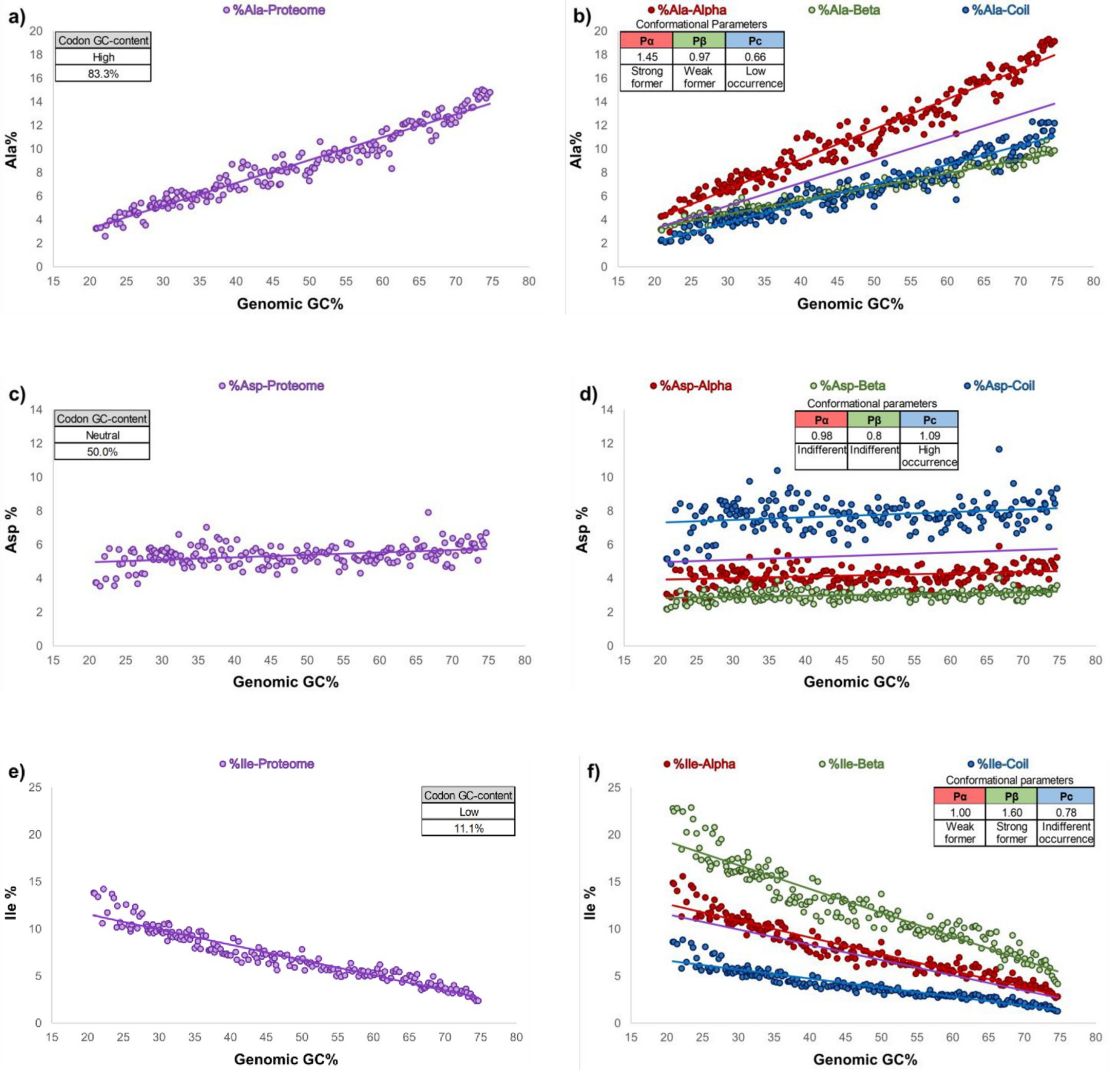
Genomic GC content bias effects on three amino acid frequencies for proteome and secondary structures of proteins. The relative frequencies of amino acids in proteomes (a, c, and e) or in the secondary structures of proteins (b, d, and f) of 192 prokaryotes are shown. The amino acids of the figures were chosen considering their tendencies to be part of each of the secondary structures of proteins (b, d, and f, for alpha-helix strong former, indifferent, or beta-sheet strong former, respectively) or considering the GC content of the codons by which they are encoded (a, c, and e, for high, neutral, and low GC content codons, respectively). As a reference, the regression lines of the amino acid frequencies obtained in the proteome analyses are shown in purple in figures b, d, and f. The conformational parameters of amino acids described by Chou and Fasman [25, 27] are shown at the top of each graphic.

As an expected consequence of the different GC contents of the codons that codify each of the 20 amino acids, it has been widely documented that the relative frequencies of the amino acids of proteomes vary as the genomic GC content fluctuates [13, 16, 17, 19, 21, 22]. In these studies, the amino acids were clustered according to the GC content of the codons that encode them, and their frequencies in the proteomes were analyzed. With increasing genomic GC content, the amino acid compositions encoded by high-GC content codons tended to increase (Ala, Gly, and Pro), while those encoded by low-GC content codons tended to decrease (Lys, Ans, and Ile). In our study, we categorized the amino acids into three groups: those with high-, neutral-, and low-GC content codons (Fig 1) and performed a similar study as that abovementioned using a set of 192 prokaryotic genomes, obtaining concordant results (Fig 3 and S1 Fig). For most of the amino acids, we observed a linear relationship between the GC content of their corresponding codons and the relative frequencies with which they are found in proteomes, while for some others, such as Met and Gln, more complex polynomial regression models better fit the curves (S1 Fig). For amino acids encoded by GC content-rich codons (Ala, Gly, Pro, Arg, and Trp; Fig 1), a positive slope of their regression lines was observed (Fig 3A and S1 Fig); amino acids encoded by neutral-GC content codons (His, Asp, Thr, Cys, Glu, and Ser; Fig 1) seem to be not importantly affected by the genomic GC content (Fig 3C and S1 Fig), while for amino acids encoded by low-GC content codons (Phe, Tyr, Lys, Asn, and Ile; Fig 1), negative slopes of their regression lines were seen (Fig 3E and S1 Fig). It should be noted that Val and Leu present unusual behavior in their slopes with respect to the group of amino acids to which they belong. That is, Val (amino acid with a neutral codon GC content) has a positive correlation with respect to the increase in genomic GC content, while Leu (amino acid with a low codon GC content) also has a positive trend (S1 Fig).

It has been extensively documented that due to the physicochemical properties of amino acids, they present specific tendencies to prevent (breaker), contribute to (formers), or have a neutral effect (indifferent) on the formation of the secondary structures of proteins [25, 27, 33–35]. In this regard, we repeated the analysis described previously for the proteome sequences but independently consider the amino acids present in alpha-helix, beta-sheet, and random coil secondary structures, which are shown as figures (Fig 3B, 3D and 3F and S1 Fig) and reported as Tables (S4 and S5 Tables). Amino acids that tend to contribute to a specific secondary structure presented higher slope values than the slope of the regression line of the proteome, while amino acids that tend to prevent such secondary structures presented lower slope values. In addition, we found that the slope values of the regression line of those amino acids with a neutral effect (indifferent) on the formation of the secondary structures of proteins were similar to those obtained in the analysis of the entire proteome. For example, the higher slope of the regression line for the secondary structure of the alpha-helix of the Ala amino acid (red line, Fig 3B) with respect to the slope of the regression line obtained by the proteome (purple line, Fig 3B) is indicative of the preference of this amino acid to exist in this type of secondary structure, while the smaller slopes for beta-sheets and random coils (green and blue lines, respectively, Fig 3B) indicate the low propensity of Ala to be part of the aforementioned secondary structures.

### Analysis of genomic GC content bias effects on the conformational parameters of amino acids in the secondary structure of proteins

**Fig 4:**
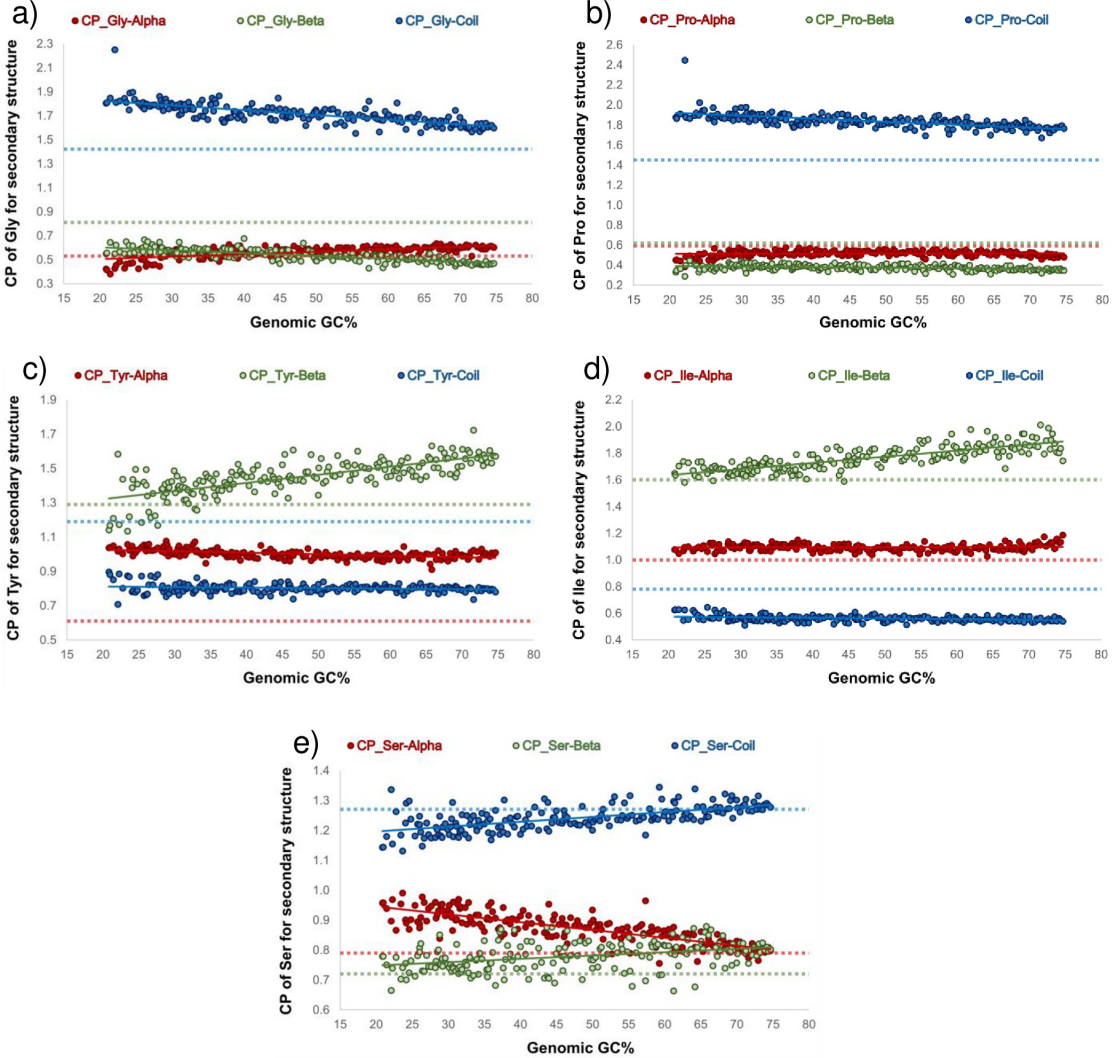
Effect of the genomic GC content on the amino acid conformational parameter values. The CP values for Gly, Pro, Tyr, Ile, and Ser in alpha-helices (red circles), beta-sheets (green circles), and random coils (blue circles) are evaluated as a function of the genomic GC content. The regression lines of the points are represented by solid lines, while the CP values reported by Chou and Fasman [25, 27] are represented by hashed lines.

The tendencies of amino acids to be part of a specific secondary structure have been characterized since 1974 by Chou & Fasman and are expressed as conformational parameters (CP) that range from 0 to 2 [25, 27]. These values were evaluated considering the frequencies of each amino acid that were present in the alpha-helices, beta-sheets, and random coils in relation to the frequencies of such amino acids in the proteome and the relative frequencies of the amino acids for each secondary structure in relation to the number of amino acids in the proteome [25, 27]. The number of proteins used in the pioneering study of Chou & Fasman was only 15. In this study, we updated these conformational values, considering the amino acid sequences of 192 full proteomes that were selected based on the GC content of their corresponding genomic sequences. Unexpectedly, we observed that some amino acids’ conformational values were not constant. They presented small but statistically significant variations depending on the genomic GC content values (Fig 4). In our analysis, we found that most of the amino acids, the conformational parameters had a regression line with a slope m <0.0047, and a *p-value* < 0.001, for each secondary structure of the proteins (S6 Table).

In accordance with our results, the amino acids whose CP values for random coils present more significant variations (given their slope and with a *p value* < 0.001) as the genomic GC contents vary are Gly and Pro for random coils (Fig 4A and 4B, respectively); Tyr, Ile, Ala, Asn, and Leu for beta-sheets (Fig 4C and 4D and S2A–S2C Fig, respectively); and Ser for alpha-helices (Fig 4E). The rest of the figures of amino acids are shown in S2 Fig, and the data from linear regression of all amino acids are presented in S6 Table.

### The genomic GC content of prokaryotic organisms imposes bias in the frequencies of secondary structures in proteomes

**Fig 5:**
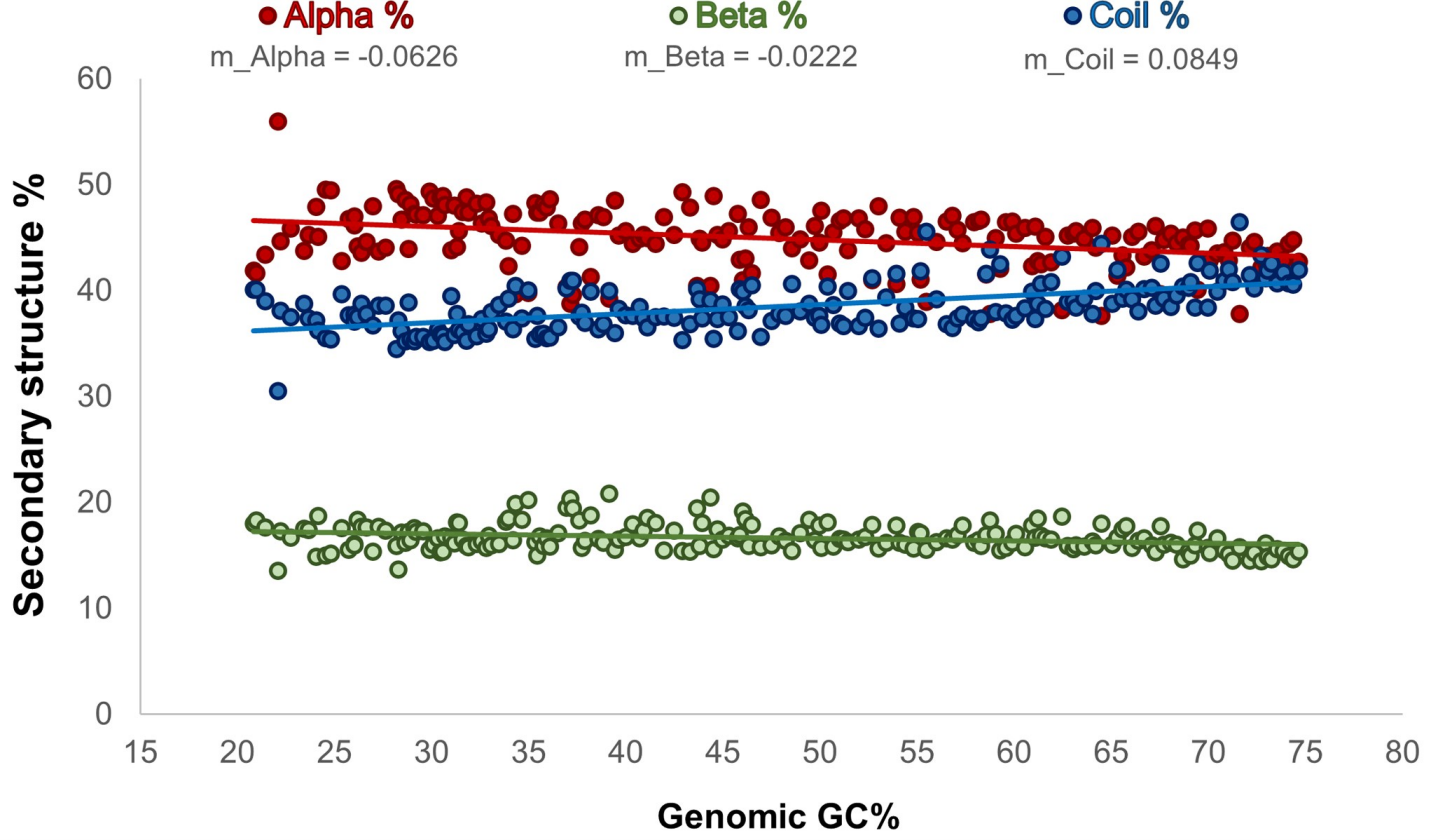
Relative frequencies of the secondary structure of proteomes as a function of the genomic GC content. The relative frequencies of alpha-helix, beta-sheet, and random coil secondary structures in our studied organisms were evaluated and plotted against their corresponding genomic GC content. A linear regression was obtained for each of the secondary structure elements. The slope value (m) for each linear regression is indicated in the figure and the linear regression with a *p-value* <0.001 is found in S7 Table.

Considering that the genomic GC content of organisms (a) may vary importantly in accordance with the phylogeny of organisms (Fig 2), (b) affects the overall frequencies of amino acids of the proteomes and in their corresponding secondary structures (Fig 3), and (c) imposes bias in the conformational parameters of the amino acid (Fig 4), here, we analyze whether the overall frequencies of each secondary structure of proteins in the proteomes vary as a function of the GC content of the genomes by which they are encoded. Our results indicate that the overall secondary structure content of proteomes is not universal but presents variations that are correlated with the genomic GC content. As the GC content of genomes increases, the relative frequencies of random coils tend to increase, while the relative frequencies of alpha-helices and beta-sheets tend to decrease (Fig 5 and S7 Table).

### GC content of genes imposes bias in the secondary structures in some families of orthologous proteins (COGs)

**Fig 6:**
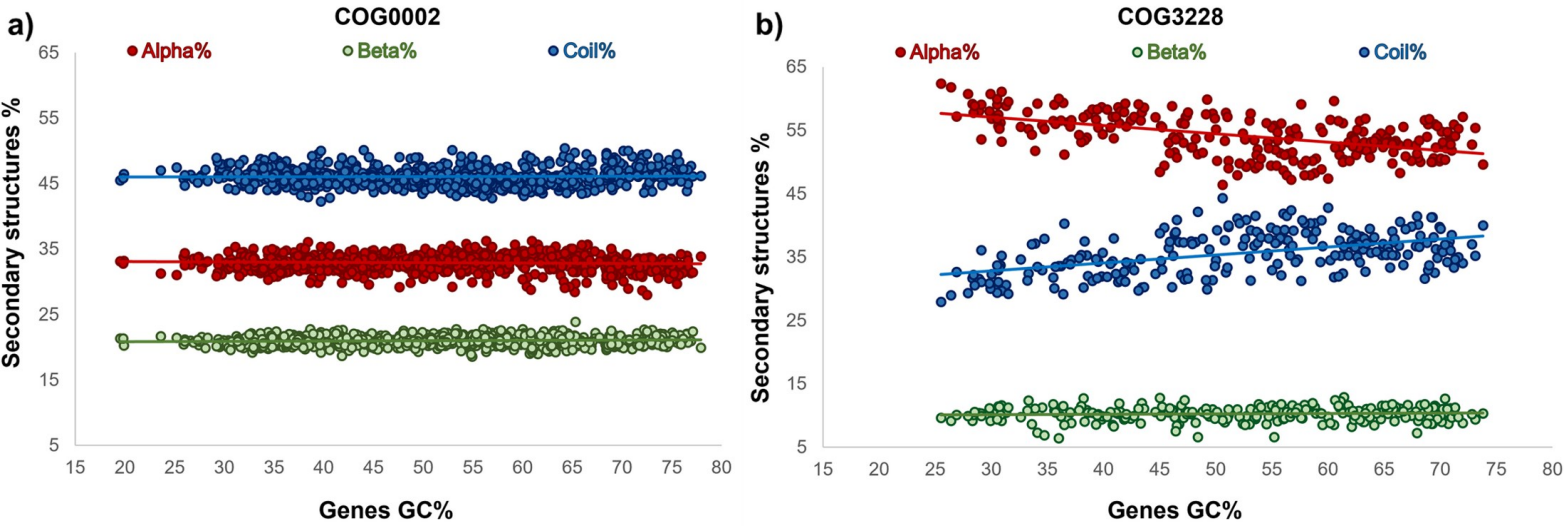
Comparative linear regression between COGs without and with bias imposed by the GC content of their respective genes. a) COG0002: Acetylglutamate semialdehyde dehydrogenase, and b) COG3228: Uncharacterized protein conserved in bacteria, respectively. The regression lines for each secondary structure are shown as solid lines. A complete set of figures is available from our GCto2D web server at https://biocomputo.ibt.unam.mx/gcto2d/.

The small but statistically significant changes in the relative frequencies of the secondary structure of proteomes (with small slope of 0.08 or minor to -0.06, but with a *p-value* <0.001 in all cases, S7 Table) as a function of the genomic GC content identified and shown in Fig 5 can be explained by at least three different reasons: (a) by the presence of different proteins in the proteomes, (b) by a change in the relative frequencies of the secondary structures in orthologous proteins or (c) by a combination of the above possibilities. With the purpose of determining which of these possibilities is the most likely to be correct, we repeated our analysis of the GC content bias effect on the secondary structure of proteins considering different sets of orthologous genes. To this end, we expanded our initial set of reference organisms to include 1,544 representative prokaryotes at the genus level. The orthologous proteins were clustered considering the COG database classification [30]. To ensure that the comparisons between proteins were mostly restricted to homologous domains, we used stringent inclusion criteria for the selection of proteins into the COG groups (see Material and Methods). The plots of the GC content of orthologous genes *versus* the relative frequencies of their secondary structures revealed that for most of the COGs, there was no significant variation in the secondary structures (with a *p-value* >0.001), as shown for COG0002 (Fig 6A and S8 Table). In contrast to this result, our study also showed that nearly 5% of the analyzed COG sequences presented small but statistically significant variations in the secondary structures of their proteins as the GC content of their corresponding genes varied. In this group, the *p-value* is <0.001 of at least two secondary structures of their proteins. One example of this type of COG is COG3228 (Fig 6B and S8 Table). The complete set of plots for all the analyzed COGs can be seen on our web page GCto2D (https://biocomputo.ibt.unam.mx/gcto2d/).

In addition to our graphical results, we represented the alpha-helices, beta-sheets, and random coils with the letters H, E, and C, respectively, and performed multiple alignments of the secondary structures of orthologous proteins. Our aligned sequences show significant conservation of the secondary structure of most of the orthologous protein groups (COGs) regardless of the important differences in the GC contents of their respective protein-coding genes, as observed for the multiple alignments of the secondary structures of COG0002 (S3 Fig). We also show the multiple sequence alignments of COG3228 as an example of orthologous proteins that presented variations in their secondary structures as the GC content of their genes varied (S4 Fig). The significant conservation for these kinds of COGs, we observed that the amino and carboxy termini of their proteins are the regions most prone to variations in the lengths and composition of their secondary structures. The full set of multiple secondary structure alignments is available on our GCto2D website (https://biocomputo.ibt.unam.mx/gcto2d/).

## Conclusion

One of the main characteristics of the genomes of prokaryotic organisms is the wide variation in the frequency at which the guanine-cytosine bases are used in their genomic DNA sequences. Pioneering studies have demonstrated the impact of genomic GC content on the relative frequency of amino acids in the proteomes of organisms [13, 16, 17, 19, 21, 22]. After several decades of these first reports, the present study goes a step further and analyzes the effect of genomic GC content on the secondary structure of proteins. Through a bioinformatic study, we found that the composition of the secondary structures of the proteomes varies in relation to the genomic GC content; random coils increase as the genomic GC content increases, while alpha-helices and beta-sheets present an inverse relationship. In addition, we demonstrate that the conformational parameters of amino acids in alpha-helices, beta-sheets, and random coils are not ubiquitous, as previously expected, but vary according to the genomic GC content. Finally, we discovered that for some particular groups of orthologous proteins, the GC content of genes imposes bias in the composition of secondary structures of the proteins for which they code.

Our research shows evidence for the plasticity of prokaryotic organisms to develop genomes with different GC contents while simultaneously encoding fully functional proteins. This can be important to include new fundamental questions in the field of evolution, protein structure sciences, microbiology, and genomics.

## Supporting information

S1 Table Amino acid conformational parameters assignments. (XLSX)

S2 Table Pipeline used in our study. (XLSX)

S3 Table Taxonomy classification of 192 prokaryotes. (XLSX)

S4 Table Lineal regression of 20 amino acids in 192 proteomes. (XLSX)

S5 Table Lineal regression of 20 amino acids in secondary structures of 192 proteomes. (XLSX)

S6 Table Lineal regression of conformational parameters in 192 proteomes. (XLSX)

S7 Table Linear regression of secondary structures of 192 proteomes. (XLSX)

S8 Table Linear regression of secondary structures of 2 COGs. (XLSX)

S1 Fig Genomic GC-content bias on the amino acid frequencies for proteome and secondary structures of proteins. (TIF)

S2 Fig Effect of the genomic GC content on the amino acid conformational parameter values. (TIF)

S3 Fig Alignment of the secondary structure elements of COG0002 according to the GC-content of their genes. (TIF)

S4 Fig Alignment of the secondary structure elements of COG3228 according to the GC-content of their genes. (TIF)
