# Supplementary figures and images for: The effect of the genomic GC content bias of prokaryotic organisms on the secondary structures of their proteins
Source: PLoS One. 2023 May 4;18(5):e0285201. doi: 10.1371/journal.pone.0285201 (PMC10159118; doi:10.1371/journal.pone.0285201)

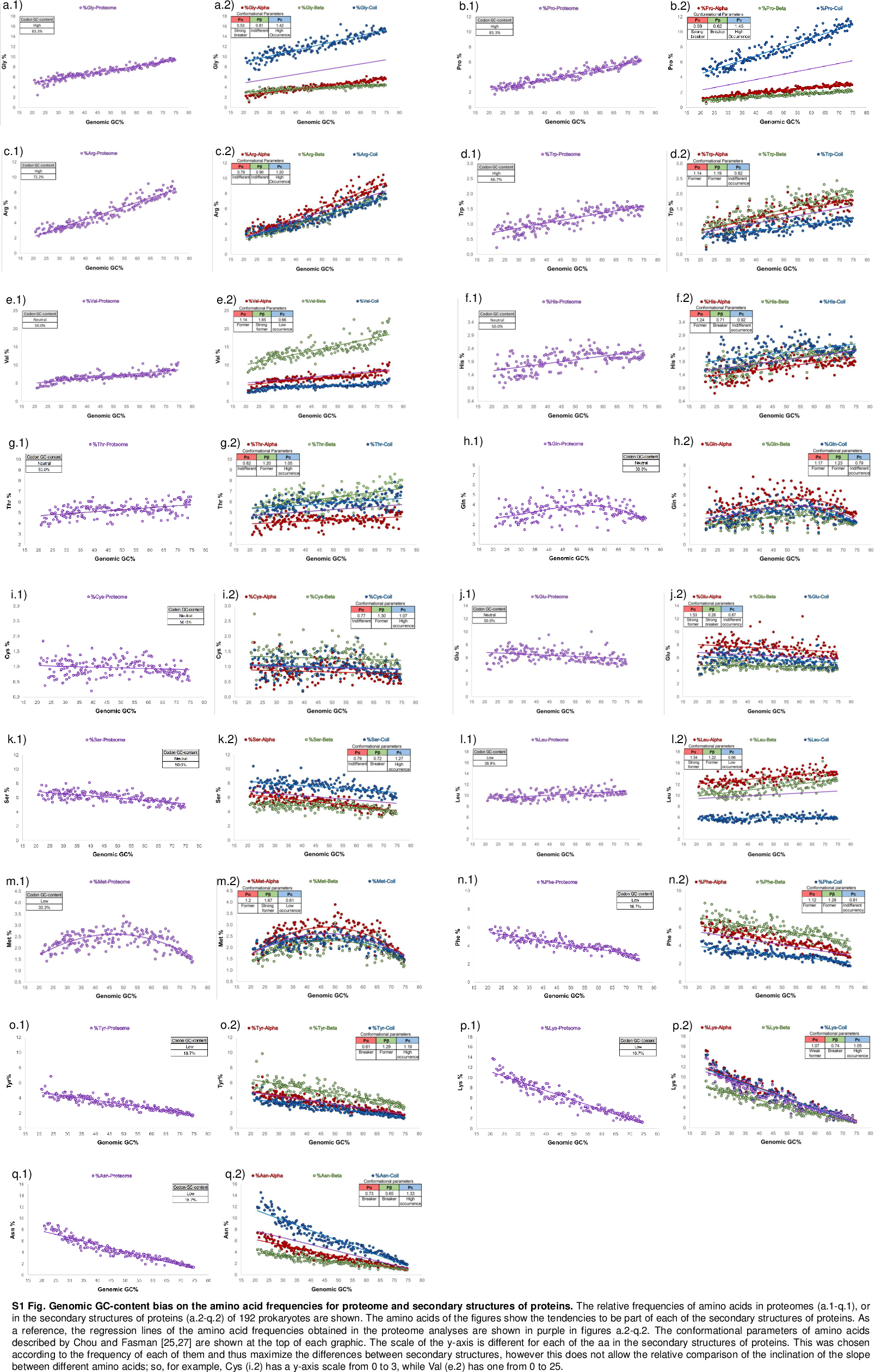

Supplement: S1 Fig — (TIF) [file pone.0285201.s009.tif]

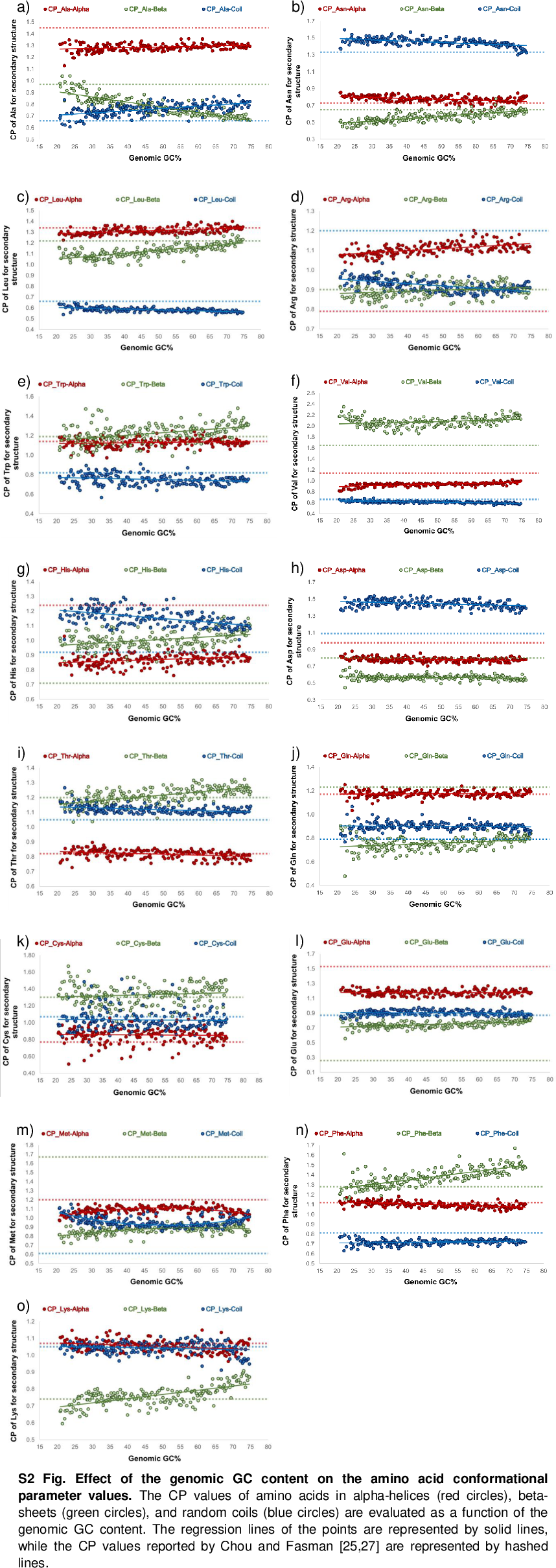

Supplement: S2 Fig — (TIF) [file pone.0285201.s010.tif]

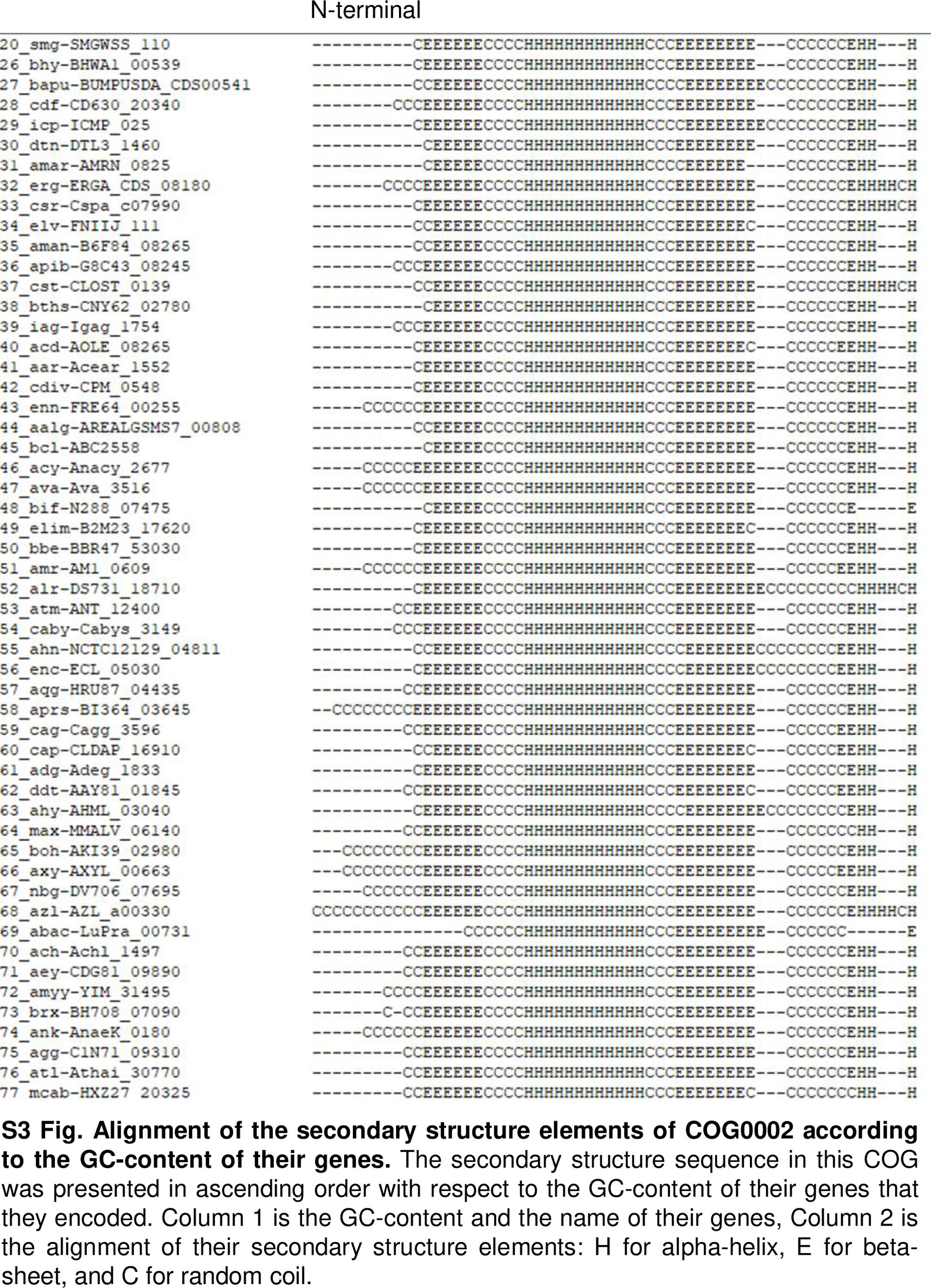

Supplement: S3 Fig — (TIF) [file pone.0285201.s011.tif]

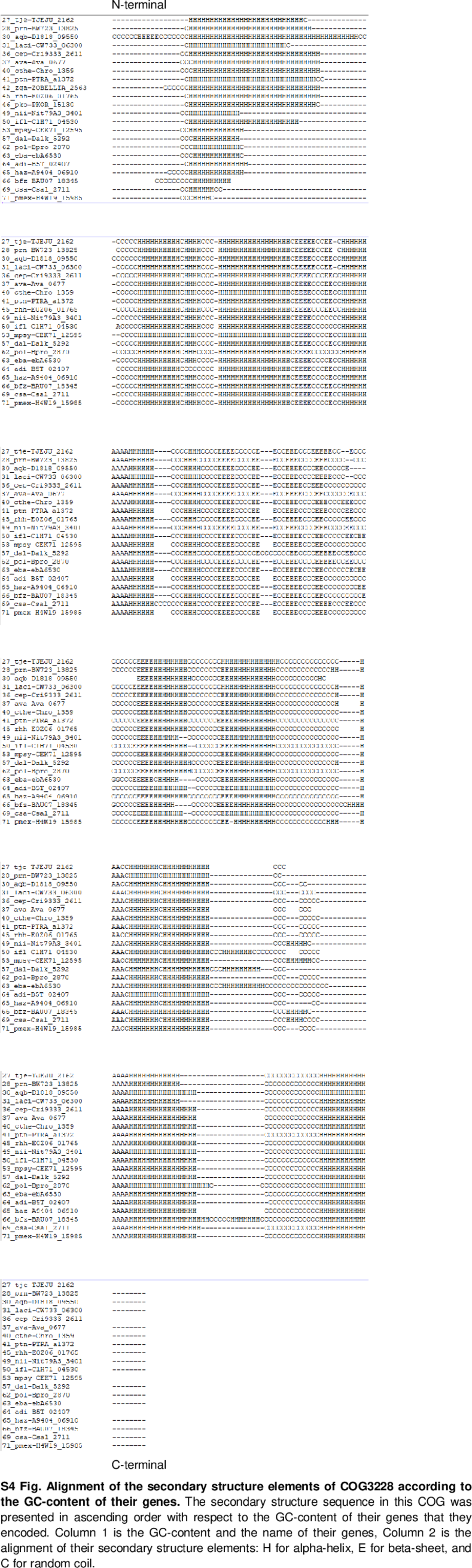

Supplement: S4 Fig — (TIF) [file pone.0285201.s012.tif]
